# Lnc MSTRG 4701.7 targets miR-1786/RORa to competitively regulate proliferation and apoptosis in chicken follicular granulosa cells

**DOI:** 10.3389/fvets.2025.1583287

**Published:** 2025-04-30

**Authors:** Chang Ma, Hengsong Wu, Chunchi Yan, Simushi Liswaniso, Xue Sun, Ning Qin, Rifu Xu

**Affiliations:** ^1^Joint International Research Laboratory of Modern Agricultural Technology, Ministry of Education, Jilin Agricultural University, Changchun, China; ^2^Department of Animal Genetics, Breeding and Reproduction, College of Animal Science and Technology, Jilin Agricultural University, Changchun, China

**Keywords:** apoptosis, chicken follicular granulosa cells, proliferation, mRNA expression, egg production

## Abstract

**Background:**

Follicular development in chickens is a highly coordinated and complex process. While the hypothalamic–pituitary-gonadal axis plays a central regulatory role in this process, the role of long noncoding RNAs (LncRNAs) remains unclear. Here, we aimed to identify key differentially expressed LncRNAs and explore their regulatory roles in follicular development, particularly focusing on the MSTRG.4701.7-miR-1786-RORa axis, to provide insights for improving fertility in low-fertility chicken varieties.

**Methods:**

Intact follicular tissues were collected from Jilin Black chickens (low-fertility group) and Lohmann Brown Laying hens (high-fertility group) at each stage of ovarian development for transcriptome sequencing to identify key differentially expressed LncRNAs for follow-up analyses. Bioinformatics analysis was also performed to determine the role of the MSTRG.4701.7-miR-1786-RORa regulatory axis, and to clarify whether that MSTRG 4701.7 targets miR-1786/RORa to competitively regulate the proliferation and differentiation of chicken follicular granulosa cells (GCs). We also knocked down and overexpressed key genes, MSTRG.4701.7 and miR-1786 and thereafter, determined changes in the mRNA and protein expression levels of axial terminal mRNA-RORa.

**Results:**

MSTRG.4701.7 promotes the apoptosis of chicken follicular GCs, while miR-1786 reverses this phenomenon. RORa was also identified as a functional target of miR-1786 in GCs, and as a competitive endogenous RNA, MSTRG.4701.7 regulated RORa expression by sponging miR-1786, thereby playing a regulatory role in GCs. Additionally, interfering with MSTRG.4701.7 expression significantly downregulated RORa mRNA and protein expression levels, while interfering with miR-1786 showed the opposite effect.

**Conclusion:**

MSTRG.4701.7 plays a critical role in follicular development in chickens by influencing GC proliferation, differentiation, and apoptosis. The resent results provide useful molecular evidence for elucidating the genetic mechanism underlying ovarian follicle development associated with egg production in chicken.

## 1 Introduction

Laying traits are closely related to follicular development and are regulated by local ovarian factors and hormones secreted by the hypothalamic–pituitary-gonadal axis. The study of key functional genes enhances understanding regarding the molecular mechanisms underlying follicular development and allows the optimization of genetic breeding. Jilin Black Chicken (JB), a meat-egg hybrid, is characterized by a high genetic diversity and shows stress resistance; however, its egg production performance is insufficient (1, 2). Therefore, studies on the molecular mechanisms underlying its egg-laying traits may be of great significance in facilitating breeding activities and improving this breed. In the present study, we aimed to provide a scientific basis for the breeding of JB chickens by analyzing the differential genes and common gene co-expression networks between JB chickens and Lohmann Brown laying hens (LB) hens, a breed with a high egg-production rate (3–5).

Long noncoding RNAs (LncRNAs) constitute an important class of noncoding RNAs (6). Recent studies have confirmed that they are closely related to poultry breeding and can play regulatory roles in this regard. Competing endogenous RNAs (ceRNAs) are involved in post-transcriptional regulation mechanisms, which constitute a major pathway by which LncRNAs participate in breeding regulation (7). LncRNAs can bind miRNAs via molecular sponge to regulate mRNA, and subsequently influence the genetic traits of animals (8, 9). Transcriptomic sequencing has also shown that MSTRG.4701.7 is a differentially expressed LncRNA between JB and LB chickens, and its targets, miR-1786 and mRNA-RORa may be related to chicken follicular development. However, the mechanism by which it regulates the proliferation, differentiation, and apoptosis of granulosa cells (GCs) remains to be clarified.

Therefore, in this study, we employed whole-transcriptome sequencing to identify key differences in lncrna-mstrg.4701.7 expression between JB and LB varieties. The molecular biological functions of the target gene, RORa and target miRNA-1786 of the LncRNA, MSTGR-4701.7, were analyzed and verified based on RNAi interference and overexpression techniques, and the molecular mechanism related to the role of MSTGR-4701.7 in follicle selection and differentiation was explored. Similarly, the regulatory effects of MSTRG.4701.7 and miRNA-1786 sponges on RORa proliferation, differentiation, and follicular apoptosis were analyzed. The co-expression network of MSTRG.4701.7-miR-1786-RORa was also constructed. The findings of this study may be of great significance in improve breeding activities as well as the fertility of JB chickens.

## 2 Materials and methods

### 2.1 Sample collection and cell isolation for culture

The LB and JB chickens used in this study were bred at the scientific research base of Jilin Agricultural University (Jilin, China). When the LB and JB chickens were 21 weeks old (average weight, approximately 1.7 kg), their average laying rates were 53 and 11%, respectively, and at 72 weeks, the average number of eggs laid per hen for LB and JB varieties were 289 and 152, respectively. At 21 weeks, 10 birds were randomly selected from each breed, euthanized via cervical dislocation, and their intact follicles were collected. Specifically, large white follicles (LWF) (diameter, 3.5–5.5 mm), small yellow follicles (SYF) (diameter, 6.0–8.0 mm), and large yellow follicles (LYF) (diameter, 8.5–10.5 mm) were collected. Following the removal of blood vessels and connective tissues, the follicles were immediately frozen and stored in liquid nitrogen for subsequent analyses.

GCs were harvested form the ovarian follicles. In brief, to isolate GCs, ovarian tissue was cut into chunks, washed with phosphate buffered saline (PBS) (Biosharp, Hefei, Anhui, China), digested with collagenase II in a water bath, and centrifuged for 5 min at 2000 rpm. The resulting supernatant was discarded and the collected cell spheres were resuspended in complete media and again centrifuged for 5 min at 2000 rpm. The supernatant is again discarded, and the GCs collected were used for subsequent analysis. Specifically, 5% × inoculated GCs were uniformly distributed in 6-well plates at a cell density of 1 × 10^6^ cells per well and incubated in 5% CO_2_ and 95% air at 37°C with saturated humidity. After 3 h, the medium was changed, and non-adherent cells were removed, while adherent cells were further incubated.

### 2.2 Transcriptome sequencing

Total RNA was extracted from follicular GCs using TRIzol reagent (Invitrogen, Carlsbad, CA, USA). To ensure high-quality analyses subsequently, RNA purity was assessed using a Nanodrop 2000 spectrophotometer (Thermo Fisher Scientific, Waltham, MA, USA). Next, RNA concentration was accurately quantified using Qubit 2.0 and quality assessment was performed using an Agilent 2,100 Bioanalyzer (Agilent, Santa Clara, CA, USA). RNA libraries were constructed from purified RNA. Thereafter, the quality and quantity of the constructed libraries were evaluated using the Agilent Bioanalyzer 2,100 system and sequencing was performed using the Illumina high-throughput sequencing platform (HiSeq/MiSeq; Illumina, San Diego, CA, USA). Further, paired-end sequencing was performed by Parsons Brinckerhoff (Shanghai, China). The raw data were quality controlled and evaluated using fastp software and the resulting filtered reads were compared with the reference genome (http://www.ensembl.org/Gallus_gallus/Info/Index) using HISAT 2. Based on the comparison results, the expression levels of the different genes were calculated. Differential gene expression, enrichment, and clustering analyses were also performed. To identify new LncRNAs, filtered reads were spliced to reduce transcript sequences and compared with known mRNA and LncRNA transcripts. Finally, differential gene expression, target gene enrichment, and cluster analyses were performed for the known and new LncRNAs.

### 2.3 Real-time PCR validation of sequencing results

Total RNA was extracted from isolated GCs 24 h after transfection using TRIzol reagent (Invitrogen). Thereafter total RNA concentration and purity were assessed using the NanoDrop2000 spectrophotometer (Thermo Fisher Scientific) and mRNA and lncRNA expression levels were determined using a Thermo first-strand cDNA synthesis kit (Sinogene, Beijing, China) with the random primers included in the kit used for cDNA synthesis. For miRNAs, reverse transcription PCR was performed in a 20-μL reaction vessel, containing 12 μL of dNANA reaction buffer and 1 μL of Real-Time enzyme, using a one-step miRNA RT kit (Vazyme, Nanjing, China) and a reverse transcription kit. The analyses were performed on a StepOne real-time PCR system (Thermo Fisher Scientific) using SYBR Green real-time PCR mix (Vazyme). Then, to verify the accuracy and reproducibility of the RT-qPCR results, we determined the transcription levels of genes such as MSTRG. Real-time PCR was also performed for RORa, miR-1786, StAR, and CCND1 according to the above method using their respective primer sequences as listed in Supplementary Table S1. The results were normalized using the 2^-ΔΔCt^ method against 18S rRNA as internal control. Different batches of follicles were analyzed in triplicates.

### 2.4 Western blot analysis

Cultured GCs were lysed using radio-immunoprecipitation assay buffer (P0013B; Beyotime, Shanghai, China) and total protein concentrations were measured using the bicinchoninic acid Assay Kit (P0010S, Beyotime) and for effective protein isolation, 12% SDS-PAGE gel electrophoresis was employed. Next, the purified protein was transferred onto polyvinylidene fluoride membranes (Millipore, Billerica, MA, USA), which thereafter, were blocked with 5% nonfat milk for 1 h at 20°C and incubated overnight with primary antibodies against RORa (Rabbit, Beyotime-AF7908, 1:1000 dilution) and *β*-Actin (Rat, Bosterbio-BM0627, 1:500 dilution). After washing with PBS, the membranes were further incubated with secondary antibodies (goat anti-rabbit IgG; cat. no. 31210;1:50,000; Thermo Fisher Scientific) at 20°C for 1 h. Next, the immunoreactive bands on the membrane were observed using UVP ChemStudio (Analytik Jena Biosolutions, Jena, Germany) and subsequently analyzed quantitatively using BandScan 5.0 software (Glyko, Novato, CA, USA). All protein levels were normalized to that of the internal loading control, *β*-actin to ensure accuracy and comparability of results.

### 2.5 Dual-luciferase reporter assay

HEK293T cells were repetitively seeded into 24-well plates at a cell density of 1 × 10^5^ cell per well. Thereafter, the cells were co-transfected with the wild-type or mutant plasmid (2000 ng per well) and either miR-1786 mimics or Numerical Control. After 48 h, luciferase activities were measured using the dual-luciferase reporter assay system (Promega, Madison, WI, USA). The miR-1786 mimics and NC used were synthesized by Juding Biotechnology Co., Ltd. (Shanghai, China).

### 2.6 Flow cytometry

The apoptosis rate of the GCs was detected using the Annexin V- fluorescein isothiocyanate (FITC)/ propidium iodide (PI) Apoptosis Kit (Solarbio, Beijing Solabao Science and Technology Co., Ltd., Beijing, China). In brief, GCs were seeded into six-well culture plates at a cell density of approximately 1 × 10^6^ cells per well and incubated for 24 h. Next, the cells were transfected with the appropriate plasmids using jetPRIME reagent (Polyplus Transfection) according to the manufacturer’s protocol. After 24 h of transfection, GCs were harvested using 0.25% trypsin (without ethylene diamine tetra acetic acid) and washed in cold PBS twice. Next, the harvested cells were stained with Annexin v - FITC (a protein-specific stain) and PI (a DNA-specific stain), and apoptotic rates were analyzed via flow cytometry (Beckman Coulter, Fullerton, California, USA) according to the manufacturer’s protocols.

### 2.7 Statistical analysis

All statistical analyses were conducted using SPSS 19.0 software (IBM, Armonk, NY, USA). Data were expressed as mean ± standard deviation. For multi-group comparison, we utilized One-way analysis of variance (ANOVA) followed by Tukey’s test and for two group comparison, we performed Student’s *t*-test. The significant difference was set at *p < 0.05 or **p < 0.01.

## 3 Results

### 3.1 Screening of key differential LncRNAs

**Figure 1:**
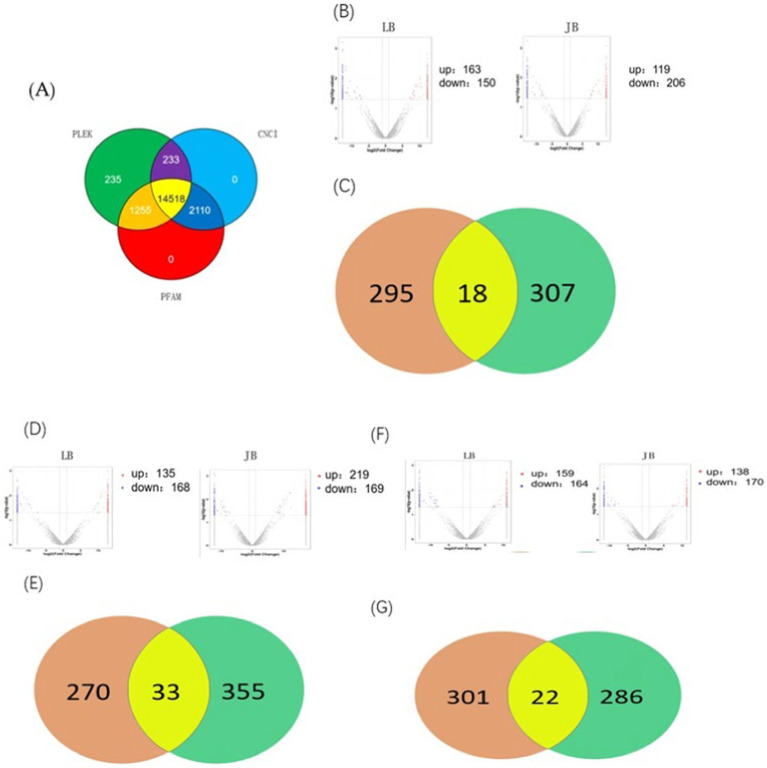
Differential LncRNAs co-expressed in JB and LB at different stages of follicular development. **(A)** Number of genes with negative potential encoded by each LncRNA based on three databases (the PLEK, CNCI, and PFAM databases). **(B**,**C)** Differentially expressed LncRNAs during the LYF and SYF stages of follicular development. **(D**,**E)** Differentially expressed LncRNAs during LWF and LYF stages of follicular development. **(F**,**G)** Differentially expressed LncRNAs during LWF and SYF stages of follicular development. JB, Jilin Black Chicken; LB, Lohmann Brown laying hens; LWF, large white follicles; SYF, small yellow follicles; LYF, large yellow follicles.

Initially, the quality of generated RNA libraries was tested. As shown in Supplementary Table S2, 18 constructed library met the quality control criteria; thus, were deemed suitable for use in subsequent experiments. Next, StringTie (https://ccb.jhu.edu/software/stringtie/) was used to assemble and compare the constructed RNA libraries using three databases (i.e., the PLEK, CNCI, and PFAM databases). Thus, we observed that the LncRNA with the highest confidence (14518) showed no coding potential (Figure 1A).

A comparison of LB and JB follicles in terms of the LWF vs. SYF stages showed 313 and 325 differentially expressed genes (DEGs), respectively, implying 18 DEGs between the two breeds (8 upregulated and 10 downregulated) (Figures 1B,C). In terms of the SYF vs. LYF stages, the LB and JB follicles showed 303 and 388 DEGs, respectively, implying 33 DEGs between the two breeds (16 upregulated and 17 downregulated) (Figures 1D,E). Further, in terms of the LWF vs. LYF stages, the LB and JB follicles showed 323 and 388 DEGs, respectively, implying a total of 22 DEGs (6 upregulated and 16 downregulated) (Figures 1F,G; Table 1). Notably, no LncRNAs were shared across the three comparison groups, while eight DEGS, MSTRG.4701.7, MSTRG. 13768.14, MSTRG. 5610.22, MSTRG. 1569.26, MSTRG. 21513.51, MSTRG. 4304.30, MSTRG. 4516.3, and MSTRG. 1569.26, were expressed in at least two comparison groups (Table 1). Since RORa, the cis-acting element of MSTRG.4701.7, may be involved in cell proliferation and apoptosis, we selected the MSTRG. 4701.7-Mir-1786-RORa regulatory axis for follow-up studies (Table 2).

**Table 2:** Common differential genes at different stages of follicular development.

ID	Log2FC	*P* value	Regulated	Group	Cis_target	Targeting miRNA
MSTRG.4701.7	Inf	0.019863347	UP	SYF VS LYF LWF VS LYF	RORa	miR-1786
MSTRG.13768.14	Inf	0.019144933	UP	SYF VS LYF LWF VS LYF	-	
MSTRG.5610.22	Inf	0.008435029	UP	SYF VS LYF LWF VS LYF	tshz3b	
MSTRG.1569.26	-Inf	0.005260387	DOWN	SYF VS LYF LWF VS LYF	-	
MSTRG.21513.51	-Inf	0.025514134	DOWN	SYF VS LYF LWF VS LYF	-	
MSTRG.4304.30	-inf	0.046814695	DOWN	LWF VS SYF LWF VS LYF	TENM4	
MSTRG.4516.3	-inf	0.006547286	DOWN	LWF VS SYF LWF VS LYF	-	

**Table 1:** Differential LncRNAs at different stages of follicular development.

Stage	Regulated	ID
LWS VS SYF	Upregulated	MSTRG.28605.14, MSTRG.22609.15, MSTRG.7577.2, MSTRG.8647.64, MSTRG.5805.13, MSTRG.11839.15, MSTRG.11584.8, MSTRG.424.10
	Downregulated	MSTRG.22025.1, MSTRG.11686.1, MSTRG.12479.8, MSTRG.2465.29, MSTRG.424.21, MSTRG.2108.38, MSTRG.28369.1, MSTRG.4516.3, MSTRG.19100.7, MSTRG.4304.30
LYF VS SYF	Upregulated	MSTRG.19100.7,MSTRG.2465.29, MSTRG.5610.22, MSTRG.22025.1, MSTRG.8859.7, MSTRG.49.19, MSTRG.16972.6, MSTRG.183.1, MSTRG.13768.14, MSTRG.18005.15, MSTRG.4701.7, MSTRG.3817.4, MSTRG.19733.2, MSTRG.49.1, MSTRG.12479.8, MSTRG.623.7
	Downregulated	MSTRG.1569.26, MSTRG.17608.9, MSTRG.62.6, MSTRG.8597.64, MSTRG.4146.26, MSTRG.8597.108, MSTRG.28426.21, MSTRG.19721.3, MSTRG.3670.6, MSTRG.21513.51, MSTRG.13098.7, MSTRG.27000.10, MSTRG.19859.2, MSTRG.28426.57,MSTRG.15485.3, MSTRG.25980.7
LYF VS LWF	Upregulated	MSTRG.13768.14, MSTRG.29803.3, MSTRG.4701.7, MSTRG.49.19, MSTRG.28184.8, MSTRG.5610.22
	Downregulated	MSTRG.9676.5, MSTRG.19511.7, MSTRG.4516.3, MSTRG.28369.1, MSTRG.12993.5, MSTRG.8597.61, MSTRG.23.18, MSTRG.21513.51, MSTRG.1569.26, MSTRG.8597.55, MSTRG.28180.5, MSTRG.22609.12, MSTRG.19282.5, MSTRG.2880.23, MSTRG.19706.6, MSTRG.4304.30

### 3.2 LncRNA MSTRG.4701.7 functions as an miR-1786 sponge

**Figure 2:**
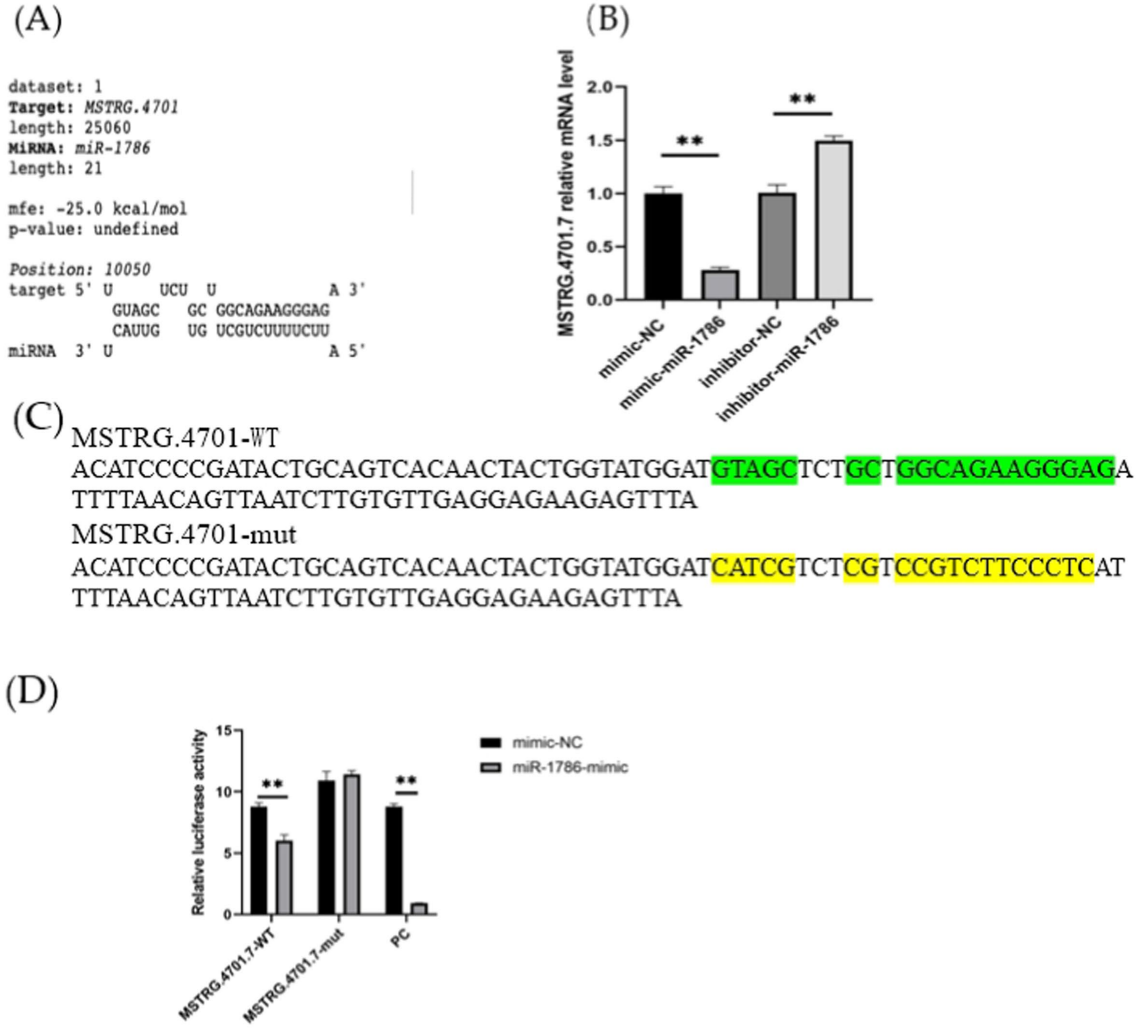
MSTRG.4701.7 functions as a molecular sponge for miR-1786. **(A)** Predicted binding site of miRNA-1786 in MSTRG.4701.7. **(B)** Changes in MSTRG.4701.7 expression level after miR-1786overexpression or knockdown. **(C)** Wild-type and mutant plasmid constructs of MSTRG.4701.7. **(D)** Luciferase activities in HEK293T cells co-transfected with miR-1786 mimics or NC controls and reporter vectors containing MSTRG.4701.7 wild-type or mutant binding sites. All experiments were performed in triplicates. Data are expressed as mean ± standard deviation (SD) of three experiments; *p < 0.05, ** *p* < 0.01. PC stands for positive control.

In this study, we investigated whether MSTRG.4701.7 functions as a sponge for miR-1786. Initially, potential binding sites between these two genes were predicted using the DIANA database (Figure 2A). Next, we constructed a dual-luciferase vector reporter system, including the wild-type (MSTRG-WT) and mutant (MSTRG-MUT) plasmids of MSTRG.4701.7 (Figure 2C). This step was followed by the co-transfection of HEK293T cells with these wild-type and mutant plasmids and either the miR-1786 mimic or negative control and the monitoring of luciferase activity. The results obtained showed that miR-1786 overexpression significantly reduced the relative luciferase activity of the wild-type construct but not that of the mutant construct (Figure 2D), suggesting that MSTRG.4701.7 is a direct target gene of miR-1786. Further, to test whether miR-1786 directly regulates MSTRG.4701.7 expression, miR-1786 was overexpressed in chicken follicular GCs. The results thus obtained showed that miR-1786 overexpression significantly downregulated MSTRG.4701.7 expression (*p* < 0.01), whereas miR-1786 knockdown significantly upregulated MSTRG.4701.7 expression (*p* < 0.01) (Figure 2B).

### 3.3 *In vitro* cell regulatory function of MSTRG.4701.7

**Figure 3:**
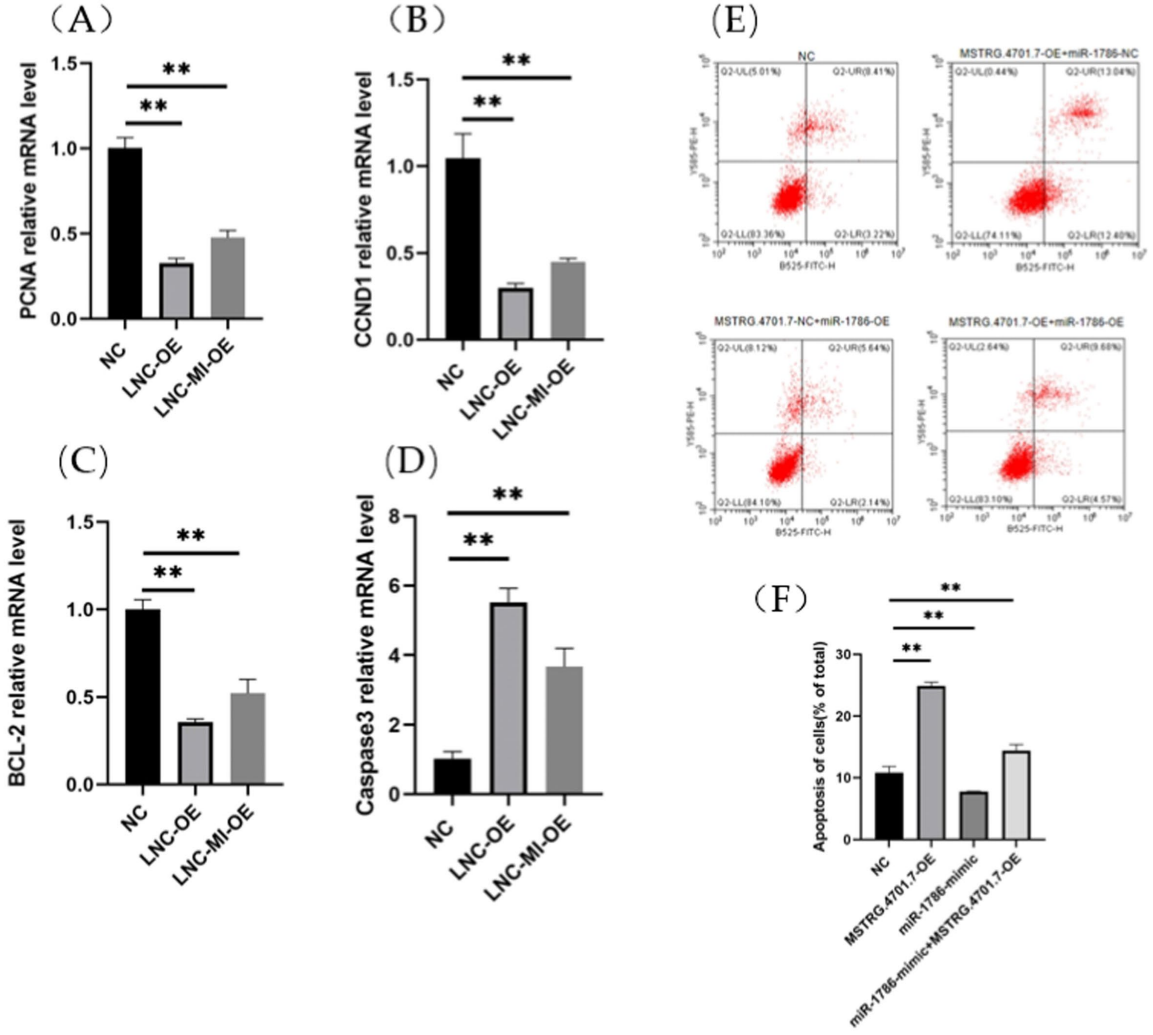
Overexpression of MSTRG.4701.7 promotes apoptosis and inhibits GC proliferation. **(A,B)** mRNA expression levels of PCNA and CCND1. **(C,D)** mRNA expression levels of BCL-2 and Caspase3. **(E,F)** Overexpression of MSTRG.4701.7 with miR-1786 to measure GC apoptotic rate. All experiments were performed in triplicates and data are expressed as mean ± SD of triplicate experiments; *p < 0.05; **p < 0.01. GCs, granulosa cells.

To further explore the effects of MSTRG.4701.7 on chicken follicular GCs, we conducted relevant functional studies on chicken follicular GCs. First, follicular GCs were extracted from chicken follicles and cultured in a thermostatic incubator. After 12 h of incubation (approximately 85% of the cells have undergone adhesion within this time), MSTRG.4701.7-OE was transfected into the follicular GCs. Experiments were also conducted to test the effect of MSTRG.4701.7 on the proliferation of chicken follicular GCs via the transfection of MSTRG.4701.7-OE or MSTRG.4701.7-OE and miR-1786-OE into the follicular GCs. Changes in cell proliferation were monitored by measuring changes in the mRNA expression levels of cell-proliferation-related genes, PNCA and CCND1. The results obtained showed that MSTRG.4701.7 overexpression downregulated PCNA and Cyclin D1 expression, implying that MSTRG.4701.7 significantly inhibited GC proliferation (Figures 3A,B). Additionally, RT-qPCR analysis revealed that MSTRG.4701.7 overexpression in follicular GCs downregulated BCL-2 mRNA expression level, while upregulating that of caspase-3 mRNA. Therefore, miR-1786 attenuated the inhibitory effect of MSTRG.4701.7 on GC proliferation (Figures 3C,D), consistent with the results of the dual-luciferase assay. Next, apoptosis experiments showed that after MSTRG.4701.7-OE transfection, the rate of apoptosis of follicular GCs increased significantly. In contrast, miR-1786 overexpression effectively lowered the effect of MSTRG.4701.7 during apoptosis in follicular GCs (Figures 3E,F).

**Figure 4:**
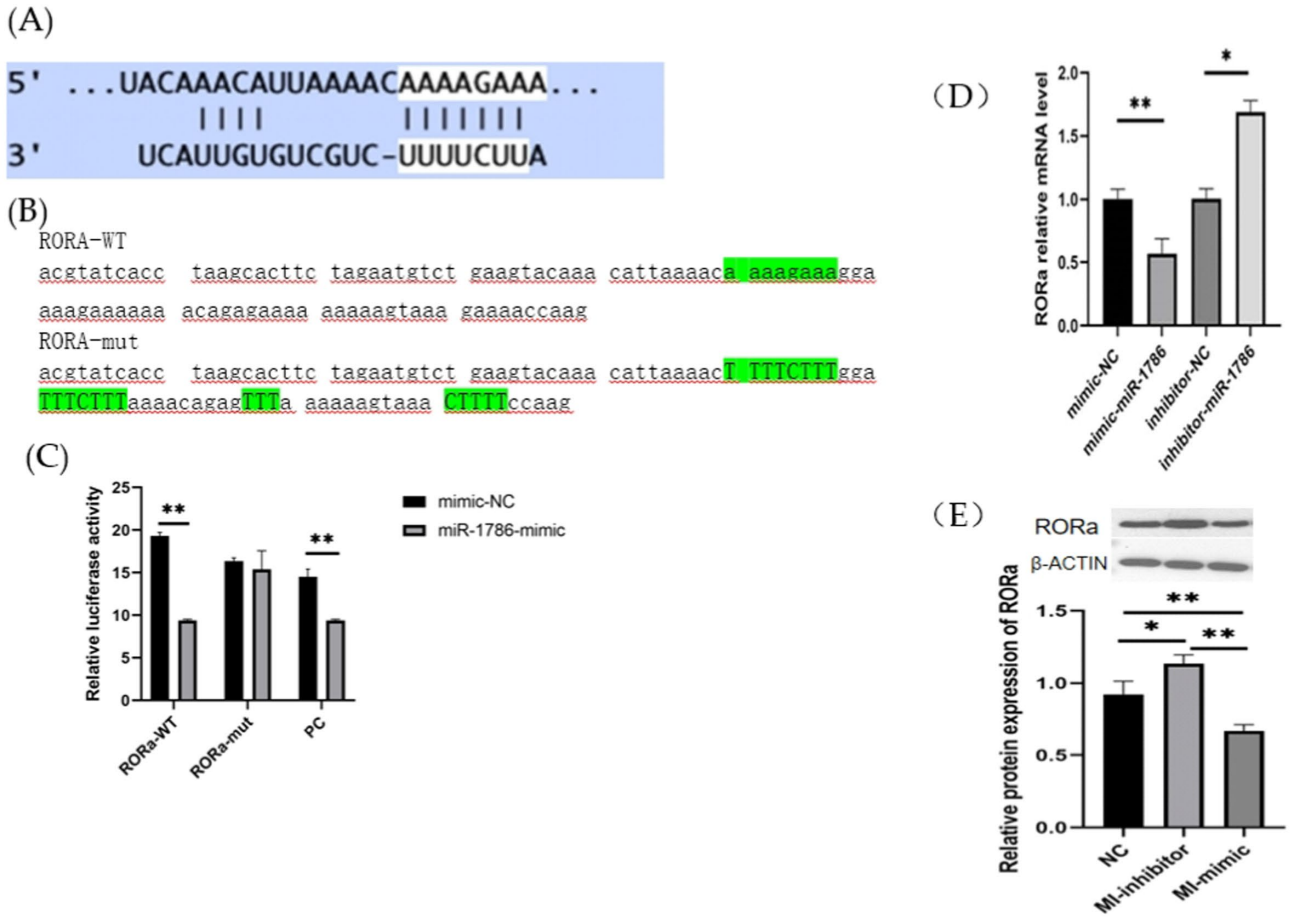
RORa is a functional target of miR-1786 in follicular GCs. **(A)** Predicted binding of miR-1786 and RORa. **(B)** Prediction of miR-1786 and RORa binding site and vector construction. **(C)** Dual-luciferase activity assay for the targeting relationship between miR-1786 and RORa. **(D)** Changes in RORa mRNA expression level following miR-1786overexpression. **(E)** Changes in RORa protein expression level after miR-1786 overexpression. Data are expressed as mean ± SD of triplicate experiments; *p < 0.05; **p < 0.01. GCs, granulosa cells. PC stands for positive control.

RORa is a functional target of miR-1786. To further elucidate its relationship with miR-1786 expression, we predicted potential binding sites for miR-1786 in the RORa transcript (Figure 4A) and subsequently constructed wild-type and mutant plasmids (RORa-mut) containing this predicted miR-1786 binding site (RORa-wt) (Figures 4B,C). MiR-1786 mimics or the miR-NC control with wild-type or mutant constructs were also co-transfected into HEK293T cells and luciferase activity was monitored. Thus, dual-luciferase reporter gene assay showed that co-transfection with RORa-wt and miR-1786 mimics significantly attenuated luciferase activity for wild-type plasmids (*p* < 0.01), but no significant effects were observed for mutated plasmids. Additionally, to demonstrate that RORa is a functional target of miR-1786, we transfected an miR-1786 interference or miR-1343 overexpression vector into follicular GCs and thereafter, performed RT-qPCR and western blot analysis. The results thus obtained (Fig S1) showed that miR-1786 overexpression significantly downregulated RORa mRNA and protein expression levels (western blotting) in follicular GCs (Figure 4D). Conversely, interference using miR-1786 inhibitor upregulated RORa mRNA and protein levels (Figures 4D,E). These results suggest that miR-1786 inhibits the expression of RORa in chicken follicular GCs.

### 3.4 MSTRG.4701.7 serves as a competing endogenous RNAs (CeRNA) for miR-1786 in GCs

**Figure 5:**
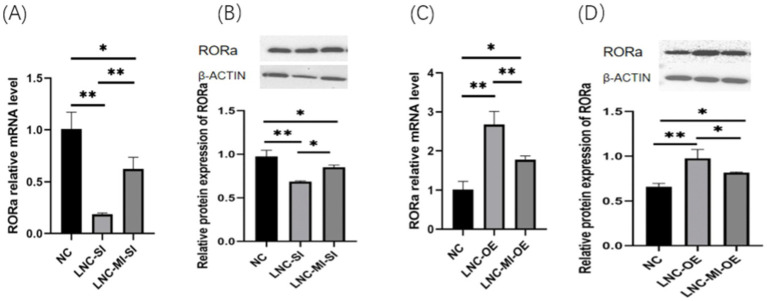
MSTRG.4701.7 regulates RORa expression by acting as a ceRNA for miR-1786. **(A)** Changes in RORa mRNA expression level after MSTRG.4701.7 co-transfection with miR-1786 interference. **(B)** Changes in RORa protein expression level after MSTRG.4701.7 co-transfection with miR-1786 interference. **(C)** Changes in RORa mRNA expression level after MSTRG.4701.7 co-transfection with miR-1786 overexpression plasmid. **(D)** Changes in RORa protein expression level after MSTRG.4701.7 co-transfection with miR-1786 overexpression plasmid. Data are expressed as mean ± SD of triplicate experiments; *p < 0.05; **p < 0.01.

To elucidate the regulatory mechanisms by which MSTRG.4701.7 modulates RORa via miR-1786 sponging, GCs were transfected with MSTRG.4701.7 and miR-1786 overexpression/interference plasmids. The results obtained showed that co-transfection with the interference plasmids of MSTRG.4701.7 and miR-1786 significantly downregulated RORa expression compared with the negative control group. Further, RORa expression was significantly higher under co-interference with MSTRG.4701.7 and miR-1786 genes than under interference with MSTRG.4701.7 only (*p* < 0.01). RORa expression was also significantly upregulated in both groups compared with its expression level in the negative control group after co-transfection with MSTRG.4701.7 and the miR-1786 overexpression plasmid. Additionally, RORa expression was significantly lower under MSTRG.4701.7 and miR-1786 co-overexpression than under MSTRG.4701.7-only overexpression (*p* < 0.01) (Figure 5).

### 3.5 RORa regulates the proliferation and apoptosis of follicular granulosa cells

**Figure 6:**
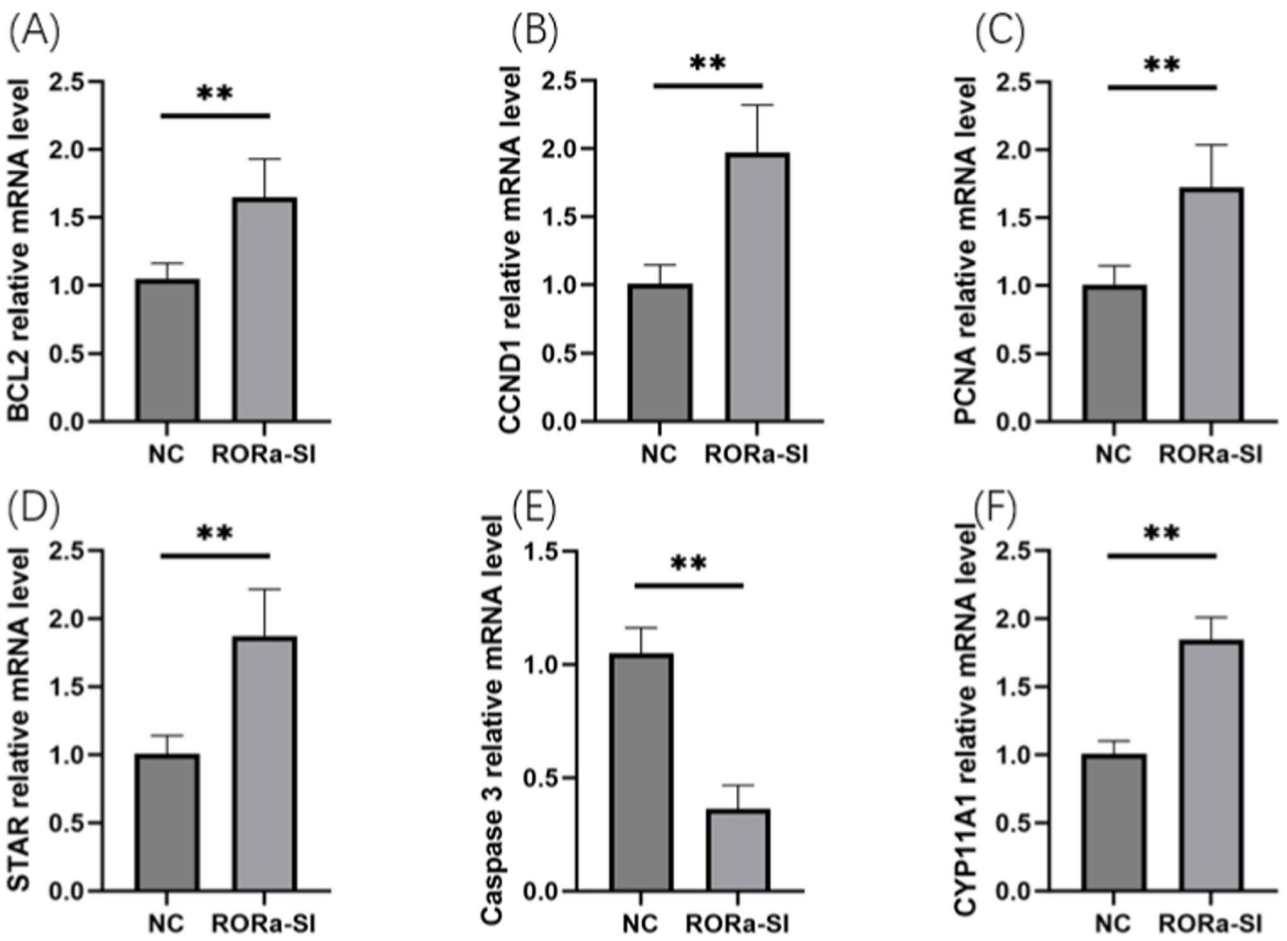
Interference with RORa can promote cell proliferation and inhibit cell apoptosis. **(A)** BCL2 mRNA expression level; **(B)** CCND1 mRNA expression level; **(C)** PCNA mRNA expression level; **(D)** STAR mRNA expression level; **(E)** Caspase3 mRNA expression level; **(F)** mRNA expression level of CYP11A1. The results were expressed as the mean ± standard deviation of the three experiments. *p < 0.05; **p < 0.01.

**Figure 7:**
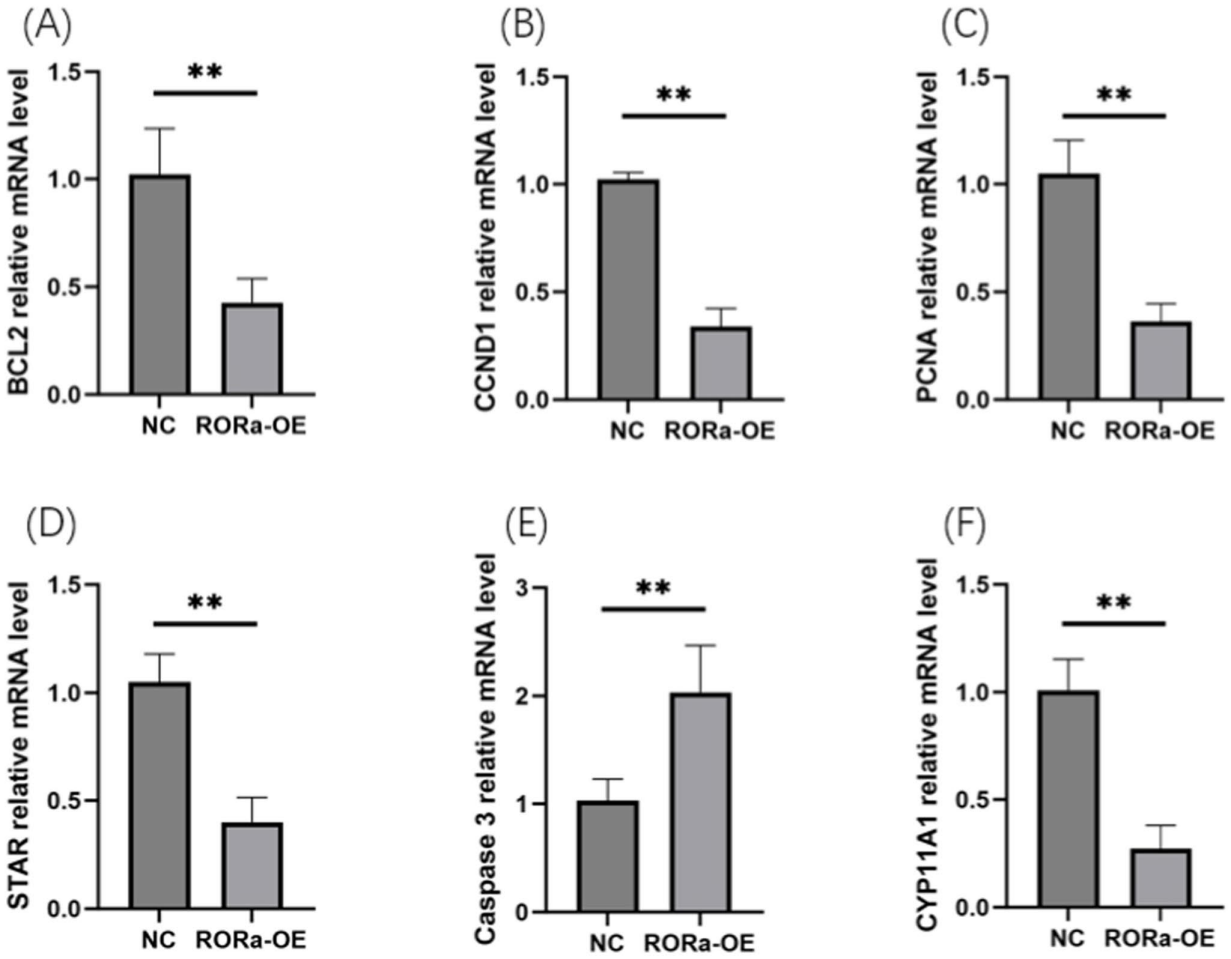
Overexpression of RORa can promote apoptosis and inhibit cell proliferation. **(A)** The expression level of BCL2 mRNA; **(B)** CCND1 mRNA expression level; **(C)** PCNA mRNA expression level; **(D)** STAR mRNA expression level; **(E)** Caspase3 mRNA expression level; **(F)** mRNA expression level of CYP11A1. The results were expressed as the mean ± standard deviation of the three experiments. *p < 0.05; **p < 0.01.

In order to investigate the effect of RORa on the proliferation and apoptosis of granulosa cells, mRNA expression levels of gene markers related to cell proliferation and apoptosis were detected by RT-qPCR. The results showed that the mRNA expression levels of PCNA, Cyclin D1 (CCND1), BCL2, STAR and CYP11A1 were significantly increased due to interference with RORa (*p* < 0.01), while the mRNA expression levels of Caspase3 were significantly decreased (*p* < 0.01) (Figure 6). Overexpression of RORa significantly decreased the mRNA expression levels of PCNA, CCND1, BCL2, STAR, and CYP11A1 (*p* < 0.01), while significantly increased the mRNA expression levels of Caspase3 (*p* < 0.01). These results indicated that RORa could promote apoptosis of follicular granulosa cells and inhibit cell proliferation (Figure 7).

## 4 Discussion

Recently, the number of studies on ceRNA has increased considerably, revealing that LncRNAs exert certain regulatory effects on follicular development in chickens via specific binding between ceRNA and miRNA (10, 11). miRNA sponges have also been shown to efficiently regulate RNA activity by competitively binding to miRNA response elements, and current studies on LncRNAs are generally based on this mechanism.

Transcriptome sequencing studies have also confirmed the involvement of LncRNAs in the regulation of follicular development in several animals, including birds (12–14). In egg-laying hens, pre-graded follicular development involves the progression of LWFs into SYFs, which subsequently enter the follicle selection stage. The selected LYFs then develop into graded follicles, implying that the LYF stage is essential for each stage of pre-graded follicle development (15, 16). In this study, LncRNA sequencing revealed that among the three stages of follicular development, LWF, SYF, and LYF, the expression levels of five LncRNAs were significantly different in LYF compared with those observed in the other two stages. This observation suggested that these five differentially expressed LncRNAs play crucial roles in the development of pre-graded follicles in egg-laying hens (17–19). Further, given that there are currently no studies on the LncRNA-MSTRG.4701.7 in relation to follicular development in egg-laying hens, we selected LncRNA-MSTRG.4701.7 for subsequent experiments.

Among the mechanisms by which LncRNAs regulate various physiological processes in cells, the sponge effect of LncRNAs on miRNA has been studied (20, 21). Using dual fluorescein reporter analysis, this study revealed that LncRNA-MSTRG.4701.7 can bind to miR-1786 through the sequence, UGUAGCUCUGCUGGCAGAAGGGAGA and inhibit miR-1786, suggesting that LncRNA-MSTRG.4701.7 can exert miR-1786 sponge adsorption effects. It has also been shown that MSTRG.5970.28 influences ANOS1expression and thus the proliferation and apoptosis of GCs via molecular sponge action and miR-133a-3p (22). LOC102163816 also induces a molecular sponge effect by targeting miRNA-miR-455-3p, promoting the expression of protein tyrosine kinase 2β (PTK2β), thereby activating the PI3K/AKT signaling pathway and regulating the proliferation of porcine follicle GCs (23). The results obtained in the present study are consistent with these previously reported findings regarding the role of MSTRG.4701.7 in follicular development.

MSTRG.4701.7 and miR1786 overexpression followed by the detection of relevant proliferation and apoptosis marker genes via flow cytometry and qPCR indicated that MSTRG.4701.7 promotes the apoptosis of pre-graded follicular GCs in laying hens, whereas miR-1786 promoted GC proliferation and inhibited the pro-apoptotic effect of MSTRG4701.7.

Subsequently, to further clarify the role of miR1786 in GCs, we used a dual-luciferase reporter assay to detect the interactions between miR-1786 and RORa. We also examined the expression of RORa by interfering with and overexpressing miR-1786. Thus, we observed that miR-1786 binds to RORa through the seed sequence (aaagaaa), downregulating RORa mRNA and protein expression. RORa is a classical signaling pathway related to cancer, and studies involving mammals have revealed that it may be involved in regulation related to cell proliferation and apoptosis (24, 25). This previously reported finding indirectly suggests a potential mechanism by which miR-1786 affects GC proliferation.

Finally, to demonstrate that MSTRG.4701.7 is a competitive ceRNA of miR-1786, we performed co-overexpression and knockdown experiments targeting both MSTRG.4701.7 and miR-1786 in GCs. The results obtained indicated that miR-1786 inhibits the up-regulatory effects of MSTRG.4701.7 on RORa but attenuated the downregulation of RORa mRNA expression owing to interference by MSTRG.4701.7. We further observed that mRNAs in the ceRNA regulatory network share common miRNA recognition elements with LncRNAs, competing for the binding of common miRNA molecules. This competition causes the mRNAs, LncRNAs, and mRNAs in the ceRNA regulatory network to balance each other’s functional effects (26–28) and thus, significantly affect each other’s expression level. Combining the dual-luciferase reporter assay and flow cytometry results, it was plausible to conclude that MSTRG4701.7 can competitively inhibit miR-1786 as a ceRNA and participate in the regulation of granulosa apoptosis.

This study further revealed that miR-1786 modulates certain interactive effects between RORa and MSTRG.4701.7. Co-transfection of miR-1786 and MSTRG.4701.7 plasmids into GCs also showed that the competitive ceRNA of miR-1786 is MSTRG.4701.7 and its inhibitory effect on RORa can be reduced via binding to miR-1786. Our findings also confirmed that the MSTRG.4701.7-miR-1786-RORa axis is associated with follicular development in chickens. MSTRG.4701.7 inhibited GC proliferation and promoted apoptosis, whereas miR-1786 exerted opposing effects. Specifically, MSTRG.4701.7 functioned as a ceRNA, inhibiting miR-1786 expression and thus, regulating RORa expression. This regulatory mechanism in GCs and its consequent effect on follicular development provides a theoretical basis for the regulation of the egg production performance of chickens from the molecular biological perspective.

## Data Availability

The datasets presented in this study can be found in online repositories. The names of the repository/repositories and accession number(s) can be found at: https://www.ncbi.nlm.nih.gov/, PRJNA669967; https://www.ncbi.nlm.nih.gov/, PRJNA670553.

## Supplementary material

The Supplementary material for this article can befound online at: https://www.frontiersin.org/articles/10.3389/fvets.2025.1583287/full#supplementary-material
