# Supplementary material for: Lnc MSTRG 4701.7 targets miR-1786/RORa to competitively regulate proliferation and apoptosis in chicken follicular granulosa cells
Source: Front Vet Sci. 2025 Apr 30;12:1583287. doi: 10.3389/fvets.2025.1583287 (PMC12075847; doi:10.3389/fvets.2025.1583287)
Supplement: Supplementary file 1 [file Table_1.doc]

**Supplementary Table S1. Primer sequences for RT-qPCR**

| Name | Primer | Sequence (5’-3’） |
| --- | --- | --- |
| 18S rRNA | Forward | 5’-TAGTTGGTGGAGCGATTTGTCT-3’ |
|  | Reverse | 5’-CGGACATCTAAGGGCATCACA-3’ |
| Caspase-3 | Forward | 5’-GAACTTCCACCGAGATACC-3’ |
|  | Reverse | 5’-GTCCACTGTCTGCTTCAAT-3’ |
| Bcl-2 | Forward | 5’-CCGCTACCAGAGGGACTT-3’ |
|  | Reverse | 5’-ACATCACGCCGCCGAAC-3’ |
| StAR | Forward | 5’-AGCAGATGGGCGACTGGAAC-3’ |
|  | Reverse | 5’-GGGAGCACCGAACACTCACAA-3’ |
| CCND1 | Forward | 5’-GCACAGCAGCACAACGTATC-3’ |
|  | Reverse | 5’-ATCTCGCACATCAGTGGGTG-3’ |
| CYP11A1 | Forward | 5'-CCGCTTTGCCTTGGAGTCTGTG-3’ |
|  | Reverse | 5'-ATGAGGGTGACGGCGTCGATGAA-3’ |
| PCNA | Forward | 5'-CTGAGGCGTGCTGGG-3’ |
|  | Reverse | 5'-ATGGCGAATGTTGCGG-3’ |
| RORa | Forward | 5'-TTTCCCTACTGTTCG-3’ |
|  | Reverse | 5'-GTATTGGCAGGTTTC-3’ |
| MSTRG.4701.7 | Forward | 5'-GGGCTGTTTTCCTTCTTACTCT-3’ |
|  | Reverse | 5'-TGACACAGCTAAACTCTTCTCC-3’ |
| miR-1786 | Forward | 5'-GCGGACGAAAAAAGACCAAA-3’ |
|  | Reverse | 5'-AGTGCAGGGTCCGAGGTATT-3’ |
