# Supplementary material for: Lnc MSTRG 4701.7 targets miR-1786/RORa to competitively regulate proliferation and apoptosis in chicken follicular granulosa cells
Source: Front Vet Sci. 2025 Apr 30;12:1583287. doi: 10.3389/fvets.2025.1583287 (PMC12075847; doi:10.3389/fvets.2025.1583287)
Supplement: Supplementary file 2 [file Table_2.doc]

**Supplementary Table S2**. Test results of total RNA quality of follicular tissue

| Sample Name | Concentration（μg/μl） | OD260/280 | OD260/230 | 28S/18S | RIN value |
| --- | --- | --- | --- | --- | --- |
| JB11 | 412.75 | 1.89 | 1.185 | 1.9 | 9.4 |
| JB12 | 520.48 | 1.857 | 1.024 | 1.7 | 9.9 |
| JB13 | 487.08 | 1.728 | 1.716 | 1.9 | 10 |
| JB21 | 1284.00 | 2.001 | 1.619 | 1.9 | 10 |
| JB22 | 788.72 | 1.941 | 1.192 | 1.9 | 10 |
| JB23 | 642.12 | 1.848 | 1.939 | 1.8 | 9.8 |
| JB31 | 499.68 | 1.832 | 1.081 | 1.9 | 9.6 |
| JB32 | 537.16 | 1.837 | 1.942 | 1.9 | 9.8 |
| JB33 | 333.90 | 1.535 | 1.469 | 1.9 | 10 |
| LB11 | 754.60 | 1.917 | 1.372 | 1.9 | 9.5 |
| LB12 | 668.52 | 1.88 | 1.12 | 1.9 | 9.8 |
| LB13 | 401.72 | 1.763 | 1.73 | 1.9 | 10 |
| LB21 | 1211.44 | 1.99 | 1.528 | 1.8 | 9.9 |
| LB22 | 690.36 | 1.86 | 1.984 | 1.7 | 9.8 |
| LB23 | 206.12 | 1.433 | 1.388 | 1.9 | 9.7 |
| LB31 | 476.84 | 1.932 | 1.425 | 1.9 | 9.5 |
| LB32 | 476.16 | 1.737 | 1.757 | 1.9 | 9.8 |
| LB33 | 535.60 | 1.634 | 1.564 | 1.9 | 10 |
